# TrialSieve: A Comprehensive Biomedical Information Extraction Framework for PICO, Meta-Analysis, and Drug Repurposing

**DOI:** 10.3390/bioengineering12050486

**Published:** 2025-05-02

**Authors:** David Kartchner, Haydn Turner, Christophe Ye, Irfan Al-Hussaini, Batuhan Nursal, Albert J. B. Lee, Jennifer Deng, Courtney Curtis, Hannah Cho, Eva L. Duvaris, Coral Jackson, Catherine E. Shanks, Sarah Y. Tan, Selvi Ramalingam, Cassie S. Mitchell

**Affiliations:** 1 Laboratory for Pathology Dynamics, Georgia Institute of Technology, Emory University School of Medicine, Atlanta, GA 30332, USA; 2 Center for Machine Learning at Georgia Tech, Georgia Institute of Technology, Atlanta, GA 30332, USA; 3 Morningside Center for Innovative and Affordable Medicine, Emory University School of Medicine, Atlanta, GA 30030, USA

**Keywords:** text mining, biomedical information extraction, biomedical literature annotation, biocuration, biomedical literature schema, named entity recognition, natural-language processing, large language model, artificial intelligence

## Abstract

This work introduces TrialSieve, a novel framework for biomedical information extraction that enhances clinical meta-analysis and drug repurposing. By extending traditional PICO (Patient, Intervention, Comparison, Outcome) methodologies, TrialSieve incorporates hierarchical, treatment group-based graphs, enabling more comprehensive and quantitative comparisons of clinical outcomes. TrialSieve was used to annotate 1609 PubMed abstracts, 170,557 annotations, and 52,638 final spans, incorporating 20 unique annotation categories that capture a diverse range of biomedical entities relevant to systematic reviews and meta-analyses. The performance (accuracy, precision, recall, F1-score) of four natural-language processing (NLP) models (BioLinkBERT, BioBERT, KRISSBERT, PubMedBERT) and the large language model (LLM), GPT-4o, was evaluated using the human-annotated TrialSieve dataset. BioLinkBERT had the best accuracy (0.875) and recall (0.679) for biomedical entity labeling, whereas PubMedBERT had the best precision (0.614) and F1-score (0.639). Error analysis showed that NLP models trained on noisy, human-annotated data can match or, in most cases, surpass human performance. This finding highlights the feasibility of fully automating biomedical information extraction, even when relying on imperfectly annotated datasets. An annotator user study (n = 39) revealed significant (*p* < 0.05) gains in efficiency and human annotation accuracy with the unique TrialSieve tree-based annotation approach. In summary, TrialSieve provides a foundation to improve automated biomedical information extraction for frontend clinical research.

## 1. Introduction

Biomedical information extraction scans text to identify structured data, including entities, relations, and events [1]. This process is vital for drug design, repurposing, and clinical meta-analysis by systematically retrieving key patient characteristics, therapeutic interventions, and clinical outcomes [2]. Leveraging and synthesizing biomedical literature accelerates drug development, drug repurposing improves treatment efficacy, and enhances evidence-based decision-making [3,4,5,6,7,8]. With over 28,000 active biomedical journals and 3000 new articles published daily, automation of biomedical information extraction is essential for extracting and synthesizing information efficiently [9].

### 1.1. Biomedical Information Extraction for PICO and Meta-Analysis

Biomedical information extraction for PICO and meta-analysis is central to the front end of clinical research. PICO is a framework used in evidence-based medicine to formulate clear, focused clinical research questions. It stands for Patient/Population/Problem (P), Intervention (I), Comparator/Control (C), and Outcome (O) [10]. By structuring questions systematically, PICO enhances the quality of systematic reviews, randomized controlled trials, and guideline development to ensure that clinical questions are specific, relevant, and answerable. Meta-analysis, on the other hand, is a statistical method used to combine and analyze data from multiple studies to derive a pooled estimate of an intervention’s effect. Clinical meta-analysis quantitatively combines multiple studies to evaluate treatment efficacy, patient outcomes, and disease risk factors [11]. However, manual methods are reliant on search filters and manual data extraction. It takes 6–10 months for a five-person team to complete a single meta-analysis [12]. Hence, automated biomedical information extraction is necessary to scale these front-end clinical research processes.

### 1.2. Challenges for Automating Biomedical Information Extraction

Natural-language processing (NLP) models offer a promising approach to automating the extraction of key biomedical data [13,14,15]. BERT (bidirectional encoder representations from transformers) models have significantly advanced biomedical named entity recognition (BioNER) by leveraging deep contextualized embeddings to capture complex linguistic patterns in biomedical texts. Variants such as BioBERT [16] and PubMedBERT [17], pretrained on biomedical literature, further enhance performance by incorporating domain-specific knowledge. Nonetheless, the complexity of biomedical language—characterized by synonyms, abbreviations, and context-dependent meanings—still poses challenges [13,15].

Biocuration, the manual annotation of biomedical text, plays a crucial role in training NLP models [14,15]. This process is highly detailed and traditionally performed by medical experts [18], making it resource-intensive. Maintaining annotation quality is essential for model reliability, necessitating the use of multiple annotators and inter-annotator agreement metrics [19,20]. The development of a well-structured and generalizable annotation schema is critical for effective manual and automated biocuration [21].

### 1.3. The TrialSieve Framework

TrialSieve streamlines biomedical information extraction through a structured annotation protocol that balances expert and non-expert contributions, enhancing scalability while maintaining quality. Expanding beyond traditional PICO methodologies, TrialSieve supports robust meta-analyses and drug repurposing by integrating NLP-based biomedical text mining with PICO principles to improve data reliability for treatment evaluation. The TrialSieve schema comprises 20 annotation categories applied to 1609 PubMed abstracts, enabling deeper quantitative, evidence-based research. An annotation user study and experiments with state-of-the-art NLP models highlight TrialSieve’s ability to improve biomedical information extraction and frontend biomedical research.

## 2. Materials and Methods

The Methods section outlines TrialSieve schema design criteria; document selection; annotator selection and training; annotation and quality control; annotator survey to evaluate the tree-based schema and annotation aids; data preprocessing; and methods related to the evaluated NLP and LLM models, which automated TrialSieve entity labeling.

### 2.1. TrialSieve Schema Design Criteria and Construction

A key contribution of the TrialSieve framework was the development of a comprehensive schema that could be utilized by both human annotators or automated models to label and extract information from published biomedical literature needed for PICO and quantitative meta-analysis. Design criteria were determined to guide the development of the TrialSieve schema and included the following:

- Schema must integrate all elements necessary for qualitative PICO and quantitative meta-analysis.
- Schema must be flexible and generalizable to any clinical domain or clinical sub-specialty (e.g., cardiology, oncology, neurology, gastroenterology, endocrinology, etc.)
- Schema must accommodate annotation of a wide variety of clinical study types, including clinical trials (phase 1, phase 2, phase 3, or phase 4), cohort study, case-control study, cross-sectional study, case study, etc.
- Schema should be optimized for biomedical information extraction from abstracts but remain sufficiently flexible for full-text structured biomedical information extraction.
- Schema design should be intuitive for annotators, including non-subject matter experts trained to do biomedical annotation.
- Schema should not require explicit identification or differentiation of the designated comparator population. Rather, the schema should enable the interventions and outcomes of all included study populations to be captured using a shared, standardized structure.
- Schema should enable the specification of global attributes relevant to the entire study versus elements that are attached to a specific patient group or arm of the study.
- Schema should enable biomedical named entity recognition using existing available NLP models.
- Schema should enable future structured biomedical information extraction for an end-to-end machine learning pipeline that extracts all elements required for performing quantitative data aggregation and statistical analysis.

The TrialSieve schema design was constructed through an iterative process that included annotators; the open-source NLP annotation software, LightTag; engineers; and clinicians. To facilitate accurate data annotation, a schema was developed through multiple iterations of feedback and refinement. The initial design comprised 14 primary tags, which were refined and ultimately expanded to 20 tags through annotation team discussions, iterative solving sessions, and quality control (QC) feedback. These refinements included the addition of new labels and the structuring of data into network graphs (also referred to as a tree-based structure) representing relationships between patient groups, drugs, dosages, outcomes, and other relevant elements. These refinements were subsequently evaluated by annotators and quality control reviewers as part of a user study.

### 2.2. Document Selection

It is well-known that many articles indexed and retrieved by PubMed for a given search will not be relevant to the research question being investigated for a clinical meta-analysis. Examples include studies that do not measure clinical outcomes, do not contain human subjects, or are not focused on a specific target disease [22]. To address this challenge, the BioSift dataset [22] was used, which presented 10,000 PubMed abstracts labeled with seven inclusion criteria well-suited to drug repurposing studies. To ensure data quality, only BioSift abstracts that met all inclusion criteria were included in the present study. Additionally, fewer than 100 abstracts deemed relevant for meta-analysis were included based on expert review.

### 2.3. Annotator Selection and Training

Seventy-two undergraduate students from a midsized university (enrollment <18,000 undergraduate students) were recruited as annotators for this project. Participants were selected from a range of science, technology, engineering, and medicine (STEM) majors, including biology, neuroscience, computer science, biomedical engineering, and chemistry. To qualify, students were required to pass two rounds of assessment: a graded annotation pre-assessment and a behavioral interview evaluating motivation. The pre-assessment utilized a simplified schema with only five labels, designed to assess candidates’ annotation aptitude prior to formal training. Candidates who scored above 68% on this assessment and demonstrated enthusiasm during the behavioral interview were accepted into the program. Ultimately, 72 students were recruited, corresponding to an acceptance rate of 64%.

Recruited annotators underwent two weeks of mandatory training, which included two 1.5-hour team-based learning sessions conducted both in-person and virtually. These sessions incorporated lectures covering the annotation schema and tools, along with collaborative annotation exercises in small groups. Training emphasized proper schema usage, annotation software proficiency, and practice annotating beta abstracts that were not included in the final TrialSieve dataset. Throughout this period, graded assessments were administered to evaluate annotators’ comprehension and accuracy.

Following the training period, annotation of TrialSieve commenced. Concurrently, annotators participated in weekly focus groups alongside peers and researchers to provide feedback on the effectiveness of the labeling schema. Based on this feedback, the schema was iteratively refined to enhance dataset quality and annotator accuracy. Throughout the project, annotators were provided with communication tools to seek support from peers, senior annotators, and project researchers. Weekly focus groups and communication support continued throughout the project duration.

In addition to the 72 annotators, 10 quality control (QC) managers were appointed to resolve annotation conflicts. These managers were required to have at least six months of prior annotation experience. Alongside conflict resolution, senior annotators were surveyed weekly to identify common annotation conflicts, monitor annotator performance, and recognize high-performing contributors. Survey findings were used to (1) inform focus group discussions and (2) provide feedback for selecting annotators for increased responsibilities, rewards, or corrective actions.

All annotators and quality control personnel were given the option to receive university credit for fulfilling an undergraduate research requirement. Those who opted out of credit were allowed to volunteer, with the flexibility to withdraw from the project at any time without penalty.

### 2.4. Annotation and Quality Control

The abstracts in TrialSieve were all labeled by 3+ annotators using the open-source annotation software, LightTag [23]. The annotators used annotation guides and open communication tools to collaborate with researchers and peers when annotating abstracts. Annotators were instructed to mark or “flag” particularly challenging abstracts or annotations made with uncertainty. These flags aided quality control managers (QC) in selecting abstracts for quality assurance review. QC managers were required to resolve all annotations that had conflicts between annotators as well as all abstract annotators flagged as challenging. The quality control protocol consisted of revising any errors in the annotations. Example revisions include misaligned text span labeling, resolving inter-annotator disagreements, and effectively defining the ground truth for a given annotation.

All annotations with no conflict and/or annotator flags were automatically accepted. On a weekly basis, the QC managers were asked to record consistently misused labels, frequently misunderstood terms, and concerns about annotator understanding or performance and submit the report to the research coordinators. Quality control reports were used to guide discussion topics in weekly annotator meetings. Finally, the QC managers were required to participate in upfront formal training and ongoing weekly meetings with the research coordinators to ensure the implementation of schema updates made during the annotator focus groups.

### 2.5. Annotator Survey to Evaluate the Tree-Based Schema and Annotation Aids

An annotator user survey was conducted to assess annotator opinions of the tree-based annotation structure and schema. Given the use of human subjects that were part of a university research course, the annotation user survey protocol was submitted to the Georgia Institute of Technology Internal Review Board (IRB) under protocol H23399. The Georgia Institute of Technology IRB protocol determined the anonymous user study to be of minimal risk and was thus “exempt” under the U.S. Department of Health and Human Services regulations for 45 CFR 46 104d.2. The study protocol H23399 was approved by the IRB on the 31st January 2024.

A 5-point Likert scale survey was developed and sent to the project annotators to explore their sentiments regarding three of the main training tools and motivational aids used in this research: (1) motivational aids, (2) relationship trees, and (3) team-learning sessions. Annotators were asked to rate a series of 32 questions using a value between 1 and 5, where 1 corresponded to “strongly agree” and 5 corresponded to “strongly disagree”. Several questions were given per category (motivational aids, 10 questions; relationship trees, 12 questions; team-training sessions, 10 questions). Of the 72 students asked to complete the survey, 38 annotators fully completed the survey, resulting in a sample size of n = 38. The survey was anonymous, and annotators were not compensated for their participation. The survey questions sought to relate annotators’ experiences with each of these tools to their willingness to make accurate annotations and their confidence in the accuracy of their annotations.

Cronbach’s alpha was used to assess the internal consistency among related groups of questions by comparing the proportion of shared variance (covariance) among survey items to the total variance observed across the survey. Cronbach alpha ranges from 0 to 1, with 0.7 to 0.95 typically deemed as optimal for retaining consistency without redundancy [24]. The Cronbach’s alpha for this survey was 0.87.

Survey responses were assessed for a normal distribution using a Q-Q plot and histogram method. A *t*-test was performed on each of the questions individually to determine if there was a difference in the sample means and an expected population mean of 3. An alpha of 0.05 was used for the statistical tests. Each question was treated as a univariate independent test.

### 2.6. Annotated Data Postprocessing

Data were post-processed following annotation and quality control. Each annotation was normalized to remove leading/trailing whitespace. Annotations made using earlier versions of the schema were discarded. Annotations from annotators whom quality control managers flagged as having consistently lower accuracy were also discarded from the final dataset. This filtering process left a total of 170,557 annotations.

Establishing consensus on span boundaries is a well-known challenge in the process of extracting evidence from clinical cohort studies [25]. A manual review of abstracts affirmed that annotators generally agreed on a large proportion of characters tagged with a specific label. However, discrepancies often occur near the boundaries of entities. In cases where multiple annotators or QC managers provided overlapping annotations, character span annotations were consolidated by adopting the majority vote label for each character. An “O” label (indicating “outside of tagged span”) was allocated to all spans without a clear majority from at least 2 annotators or QC personnel. This approach proved to be the most efficient in resolving discrepancies and maintaining high-quality span annotations. Annotators were instructed to annotate entire words; therefore, word breaks were not observed when aggregating at a character level.

Abstracts with fewer than 5 distinct span annotations after postprocessing were flagged for not having sufficient data and were removed. After aggregating, postprocessing, and removing low-annotation abstracts, the resultant dataset had 52,638 distinct, non-overlapping, high-quality span annotations and 1609 abstracts. These abstracts and annotations were used to train and evaluate the included models.

### 2.7. Models to Automate TrialSieve Entity Labeling

Four BERT-based models were utilized for this study: BioBERT (dmis-lab/biobert-base-cased-v1.2); BioLinkBERT (michiyasunaga/BioLinkBERT-base), KRISSBERT (microsoft/BiomedNLP-KRISSBERT-PubMed-UMLS-EL), and PubMedBERT (microsoft/BiomedNLP-PubMedBERT-base-uncased-abstract-fulltext). Each model was pretrained on large biomedical corpora and was selected for high performance on other biomedical named entity recognition tasks. All models were fine-tuned on our dataset for the named entity recognition task in TrialSieve.

This work primarily centered on NLP-based models. Nonetheless, one large language model (LLM), GPT-4o, was also included in the evaluation. It was chosen as a representative LLM candidate based on its success in prior foundational work examining biomedical information extraction tasks [14].

#### 2.7.1. Evaluation Metrics

The performance of these models was evaluated based on four standard metrics used for named entity recognition: accuracy, precision, recall, and F1-score. The evaluation was performed on a held-out test set comprising 15% of the abstracts.

#### 2.7.2. Implementation Details

Tokenizers and pretrained weights of all models were downloaded from Huggingface at the time of model training. Abstracts longer than 500 tokens were split along sentence boundaries into chunks with fewer than 500 tokens. A predefined split of 70% of abstracts was used for training and 15% each for validation and testing. Each model was trained for 100 epochs using a batch size of 128. Parameters were optimized using AdamW [26] with a learning rate of 0.0005. A linear decay learning rate schedule was implemented, with the first 20% of training epochs designated as a warmup.

## 3. Results

**Figure 1:**
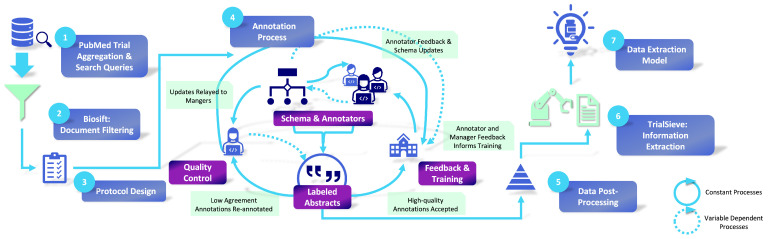
Flow of data through TrialSieve annotation and modeling process. Data was initially retrieved from PubMed and filtered down to relevant abstracts using BioSift [22]. Data was then distributed to student annotators. A subset of data was used for quality control, and annotations were used in weekly meetings to illustrate and correct common errors. Annotations were then aggregated through a postprocessing pipeline and used to train machine learning models.

**Figure 2:**
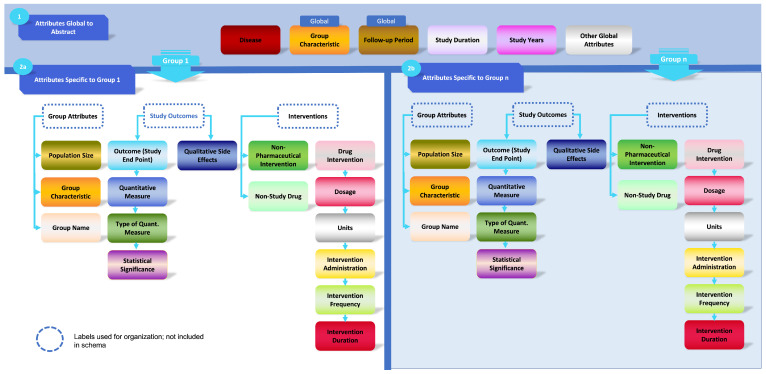
TrialSieve Annotation Schema. This figure details how annotations would be organized in a study with two population groups, e.g., an intervention and a placebo. Horizontal columns indicate annotations that, when related, are connected to provide easily parsed details on characteristics, interventions, and outcomes of each group studied. Additional study-wide characteristics are included as global characteristics.

This study introduces, TrialSieve, an assortment of 1609 abstracts annotated for biomedical entity recognition. TrialSieve is aimed at identifying clinically significant elements crucial for drug repurposing clinical meta-analysis. The comprehensive process of abstract selection, annotation, and quality control is illustrated in Figure 1. The selection criteria for these abstracts were formulated in partnership with licensed clinicians to ensure the labeled information was pertinent to drug repurposing and pharmacovigilance. Abstracts were preemptively screened to ensure that annotated abstracts contained relevant clinical data [22]. Specifics regarding these criteria and other dataset characteristics are provided in Figure 2. Each abstract was annotated by at least three annotators. Expert senior annotators cross-checked a subset of inconsistently labeled abstracts during a quality assurance phase.

### 3.1. TrialSieve Schema: A Tree-Based Approach for Accurate, Comprehensive Annotation

The final schema, illustrated in Figure 2, consists of 20 text labels and introduces a hierarchical categorization strategy that addresses prior annotation limitations. It was designed to encompass all necessary fields for conducting both qualitative PICO and quantitative meta-analyses using published clinical trial and cohort study records. Importantly, the schema is sufficiently detailed to capture key variables while maintaining generalizability across diverse clinical domains. Consequently, the TrialSieve schema is not restricted to a single disease, outcome, or pharmaceutical class. To ensure its applicability in real-world clinical settings, the schema and annotation protocol underwent review by clinicians from the Morningside Center for Innovative and Affordable Care at Emory University (see Acknowledgment Section).

#### 3.1.1. Global Versus Group Attributes

Briefly, the TrialSieve schema (Figure 2) specifies attributes based on properties that apply globally to all parts of the study abstract versus properties that only apply to one or more specific groups (or arms) of the study. Global attributes include a group characteristic shared by all groups in the study, the follow-up period, and other global attributes, which are specified by the annotator. The group-specific attributes are applied to each patient group or population. The tags include three tags from the abstract level (disease, study duration, study years) and 17 tags shown in Level 2, which are divided into 3 branches: group attributes that define the population, study outcomes that define and quantify the types of measure used to assess outcomes, and study interventions that specify the drug(s), intervention(s), or methods(s) the population received.

Beyond biomedical entity annotation, the schema incorporates relationships between spans, allowing for the organization of patient groups following the same treatment protocol. Each patient group is structured as a tree consisting of two node types: (1) tagged spans, representing text annotations, and (2) pseudo-nodes, which serve as organizational elements without direct text correspondence (e.g., “group”, “subgroup”). The root of each tree is a “group” pseudo-node to which sample size and population characteristics are attached. Interventions—including pharmaceutical and non-pharmaceutical treatments—are linked to this node, with drug treatments further associated with dosage, route of administration, frequency, and duration. Outcomes and adverse effects are connected to their respective quantitative measurements, measurement types (e.g., odds ratio, count), and statistical significance. The complete set of tags and relations is depicted in Figure 2.

#### 3.1.2. Example Application of the TrialSieve Schema

**Figure 3:**
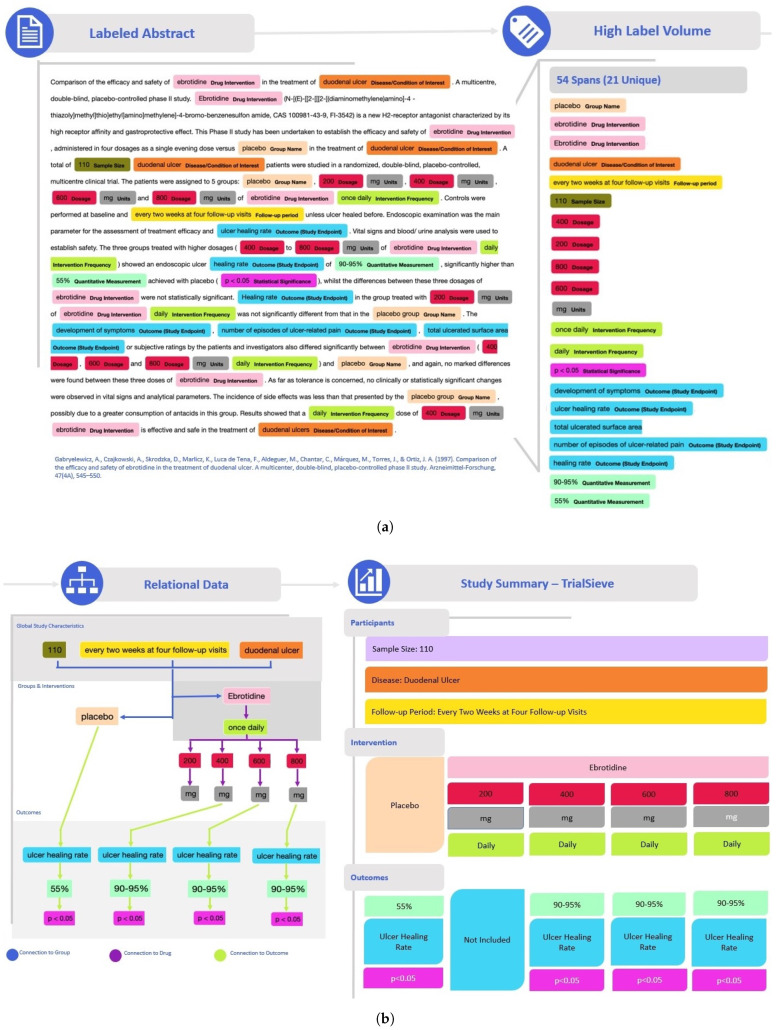
Example application of the TrialSieve schema and data annotation framework to an annotated biomedical abstract from a study published by Gabryelewicz et. al. [27] (**a**) The abstract is tagged using the TrialSieve schema; 54 spans are identified to have 21 unique labels. (**b**) Data are mapped to a relational database (**left**). A study summary (**right**) visualized key extracted quantitative outcomes and labels necessary for meta-analysis.

Figure 3a shows a detailed example abstract from a study published by Gabryelewciz and colleagues [27] that was annotated using the TrialSieve schema. The color-coding matches the schema tree shown in Figure 2. Notice there are 54 spans, including 21 unique spans, labeled in this abstract, which collectively enable all biomedical information extraction required for quantitative data analysis.

Figure 3b shows the tree-like nature that compares the two populations from the duodenal ulcer study of Gabryelewicz and colleagues [27]. This study contained two main patient populations, which received either a placebo and or the study drug of interest, ebrotidine. The left side of Figure 3b shows the tree-like relational data structure used to annotate both groups. The right side shows the study summary with the three main levels (participants, intervention, outcomes). Note that the TrialSieve schema does not explicitly state the comparator group. Rather, each group within the study has the same tagging structure available. This design choice avoids the ambiguity of either a human or a computer algorithm having to choose a single comparator group in studies with multiple arms. Moreover, it makes the schema more flexible for future large-scale automated biomedical information extraction.

The first main level of the tree illustrates the population size was 110 patients (olive color), the frequency of the follow-up period (shown in yellow), and the primary disease being examined (shown in orange), duodenal ulcer. The second main level branches into the arms of the study. On the left was the comparator group, which was the placebo (shown in grey) that was given no intervention. On the right was the study intervention group, which was given ebrotidine (shown in light pink). The study intervention drug arm records the frequency of the intervention (shown in lime green), the quantitative dose (shown in red), and the dose units (shown in grey). Since the comparator group, placebo, was given no intervention in this example abstract, there were no additional intervention details recorded. In this example, the third main level of the tree contains the study outcome characteristics, which include the specified outcome of ulcer healing rate (shown in aqua). Finally, the quantitative *p*-value significance reported by the authors is tagged (shown in magenta).

Figure 2 not only shows the schema, but it also represents the relationship trees completed by annotators as part of the annotation process. Each node in the relationship tree constitutes a label in the annotation schema, and the connections between nodes constitute the relationships applied between entities. The relationship tree represents a template structure that is repeatable and can be applied to most texts. Student annotators were expected to learn the tree structure and annotate texts by finding the appropriate entities in the text and filling in the nodes of the tree based on these entities. Trees were allowed to be pruned for texts missing some of the template nodes or expanded depending on the number of different independent entities provided in the text. The use of visual tree-based completion as the key process for annotation differed greatly from traditional written rule-based only protocols [18,28,29].

### 3.2. Dataset Statistics

The statistics for the dataset annotated using the TrialSieve framework are presented in Table 1. The final annotated dataset consists of 1609 abstracts, 170,557 annotations, and 52,638 final spans. The user-driven TrialSieve schema facilitated a more intuitive annotation process for clinical trial records, which often contain complex terminology. Utilizing undergraduate students instead of time-constrained subject matter experts (e.g., licensed clinicians) enabled the curation of a larger number of sample abstracts. Incorporating user feedback into the schema design enhanced both annotation accuracy and inter-annotator agreement. Additionally, the annotated labels improved the tagging of non-contiguous text spans, allowing compatibility with annotation software that assigns a single label per text span.

### 3.3. Automated Model Performance

Each of the models (BioLinkBERT, BioBERT, KRISSBERT, PubMedBERT, GPT-4o) was evaluated to determine the ability to detect entities in TrialSieve. Table 2 presents the overall results of the evaluation, including accuracy, precision, recall, and F1-score. Among the evaluated models, BioLinkBERT demonstrated the highest performance in terms of accuracy (0.875) and recall (0.679). PubMedBERT scored the best on precision (0.614) and F1-score (0.639). Both BioLinkBERT and PubMedBERT outperformed BioBERT and GPT-4o across all metrics. BioBERT had the lowest accuracy (0.808) and precision (0.468), whereas GPT-4o had the lowest recall (0.459) and F1-score (0.506).

To gain deeper insights into entity-specific performance, we analyzed the results for each entity category, as shown in Table 3. Notably, PubMedBERT consistently achieved the highest precision, recall, and F1 scores for most entity categories. Importantly, different models showed varying strengths and weaknesses in entity detection. For instance, BioBERT demonstrated higher performance in the follow-up period, while KRISSBERT achieved better results in Intervention Frequency. When examining solely the traditional PICO elements, the TrialSieve schema resulted in similar performance as previous 2-stage NLP pipelines that use both sentence classification and named entity recognition with BERT-based models [30].

### 3.4. Error Analysis

**Figure 4:**
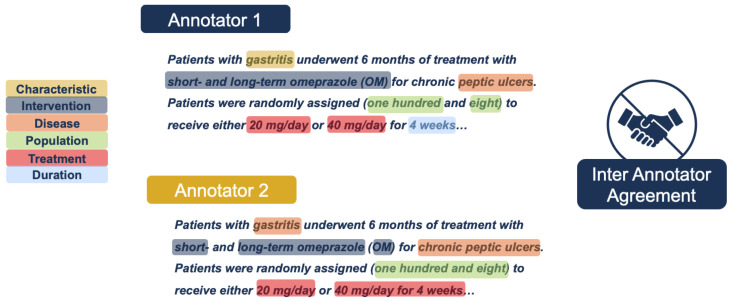
Simplified example of hypothetical labeling differences between two different annotators due to dissimilar perspectives of the biomedical text. Annotator One perceived ‘gastritis’ as a disease ‘Characteristic’, according to the predefined schema, while Annotator Two assumed ‘gastritis’ was the primary ‘Disease’ in the text. Annotator One also classified the primary ‘Disease’ in the text as ‘Peptic ulcers’, while Annotator Two included the word ‘Chronic’, making these annotations dissimilar and ‘wrong’ in the eyes of the model. Also, notice the differences in how spaces are highlighted and/or tagged.

Enhancing annotation accuracy presents a significant challenge due to the inherent variability in how different annotators may interpret and label the same text. Inter-annotater agreement is one way to assess variability. Inter-annotator agreement (IAA) is a measure of consistency between multiple annotators when labeling the same data, reflecting the reliability and reproducibility of the annotation process. See Figure 4 as a simplified example of inter-annotator disagreements. Traditional annotation projects typically rely on predefined rules and guidelines to mitigate low inter-annotator agreement. However, it is impractical to account for all possible scenarios that may lead to classification discrepancies. Furthermore, research suggests that excessively detailed guidelines do not necessarily improve annotation accuracy, as their complexity and time-consuming nature reduce the likelihood of consistent adherence [28].

An error analysis was conducted on the human-annotated data and one of the top-performing models, BioLinkBERT. In this process, highly experienced annotators re-examined a randomly selected subset of 237 abstracts annotated by humans and BERT.

An annotation is considered an error if any of the following four situations occur:

- **Wrong label:** The assigned label is incorrect.
- **Wrong overlap:** The tagged text is either too short, missing important information, or too long, including unnecessary words.
- **Multiple entities:** The tagged text includes more than one entity.
- **Invalid tag:** The text was tagged when it should not have been.

Table 4 summarizes the details of errors in the re-examined subset compared to BioLinkBERT. The BERT-based model correctly annotated 71.48% whereas humans correctly annotated 50.35%. The biggest difference between humans and the BioLinkBERT model trained with a portion of the TrialSieve dataset was the span length. However, evaluation of the accuracy results for the re-examined subset requires careful consideration of context, namely error type.

Table 5 presents the percentage of errors made by human annotators and BioBERT across four classified error types. Humans were more prone to errors in span selection, either under-selecting or over-selecting entity boundaries—categorized as ’wrong overlap’ in the table. These span-based tagging errors primarily stemmed from annotation inaccuracies when using a mouse, often resulting in extra whitespace at the end of an entity tag or the inadvertent truncation of a span.

Additionally, during this re-evaluation, only previously annotated spans were assessed, while missed spans—text that should have been tagged but was overlooked—were not considered. If these omissions were taken into account, human performance would lag even further behind BERT. Human annotation was not only less precise but also covered 20% fewer spans than BERT.

Despite the inherent noise in manual human annotation, the error analysis indicates that NLP models trained on such data can match or, in most cases, surpass human performance. This finding highlights the feasibility of fully automating biomedical information extraction, even when relying on noisy human-annotated datasets.

### 3.5. TrialSieve User Study: Annotator Opinions on the Tree-Based Approach and Annotation Aids

This study introduces a novel visual, hierarchical annotation structure—relationship trees—designed to enhance the learnability of the annotation schema and improve annotation consistency, thereby increasing inter-annotator agreement (IAA) and accuracy. To assess the effectiveness of the TrialSieve framework, a user study was conducted to evaluate annotator perceptions of three key novel components.

- Annotator perceptions of relationship trees were evaluated regarding their effectiveness in facilitating schema learnability and enhancing annotation accuracy.
- The influence of motivational aids, including awards, course credit, and perceived initiative value, on annotator accuracy and motivation was assessed.
- Annotator perspectives on the impact of team-training sessions on annotation accuracy and inter-annotator agreement were examined.

The voluntary, anonymous survey had ten questions with statistically significant results. Five of these questions discussed the use of relationship trees in the curation of biomedical texts. Four of the significant questions concerned motivational aids, and only one question with significant results concerned team-based training sessions (see Table 6).

Annotators indicated that the most important motivators for producing accurate annotations were letter grades, awards, and the opportunity to be promoted to a management role on the project. The most de-motivating factor was the experience of any technical issues with the LightTag annotation software, which impeded their ability to annotate.

The results indicate a high preference for the usage of relationship trees and a high degree of perceived accuracy while using relationship trees rather than conventional annotation methods. Questions such as “I found that using relationship trees made it easier for me to label text accurately”, “I believe I may have made LESS errors when I used relationship trees when annotating”, and “Using relationship trees improves my ability to annotate accurately”, all express the same sentiment, indicating that annotators feel more confident about annotations made using relationship trees and that the use of this tool aids annotation accuracy.

The only significant result from the team-based learning section of the survey indicated that students feel that collaborating with peers during annotation training is a useful learning tool; however, compared to the other two categories, team-based training sessions seem less influential in the eyes of annotators.

## 4. Discussion

Prior datasets for biomedical information extraction automation have largely remained private and/or have not utilized a schema that labels quantitative information necessary for downstream analysis common in drug repurposing and design. The TrialSieve framework, including the TrialSieve schema and corresponding annotation protocol, was used to construct a large, open-source annotated dataset consisting of 1609 PubMed abstracts. Each TrialSieve abstract was annotated by 3+ annotators. Thus, TrialSieve is suitable for generalizable, structured quantitative biomedical information at scale, whether by humans or NLP automation.

### 4.1. Role of the TrialSieve Tree-Based Schema in Improved Biomedical Information Annotation

TrialSieve was developed through an iterative process that incorporated relationship trees to create a more flexible and generalizable schema. This approach enables the extraction of labeled, quantitative data for clinical meta-analysis and drug design. The user study on TrialSieve demonstrated that annotators found that relationship trees significantly improved both the ease and precision of annotation. In particular, this advantage persisted even among annotators initially trained on an earlier, non-tree-based annotation protocol before transitioning to the final tree-based schema TrialSieve.

Studies suggest that visual annotation schema, such as tree-like structures with nodes linked to specific elements, can aid text categorization [28]. While slight improvements in label accuracy were observed, overall annotation accuracy and inter-annotator agreement did not significantly improve. In particular, the study by Langer and colleagues involved only two annotators with prior experience in traditional annotation methods, which may have limited the impact of the tool on skill acquisition or statistical significance [28].

In biological text analysis, orthogonal neighboring trees have improved visualization [31]. Similarly, in the medical field, structured visual representations such as treatment trees improve the comprehension of complex information [29,32,33]. Studies indicate that doctors and patients benefit from visual aids over numerical or textual data [32,33]. Learning clinical concepts is challenging, even for medical students, but audiovisual tools enhance motivation, engagement, and concentration [34,35].

### 4.2. TrialSieve Dataset Compared to Prior PICO Datasets

A range of corpora annotated with PICO elements across abstracts and full-text articles have been assembled over time. However, the majority are still not publicly accessible. One such corpus was generated by Kiritchenko et al. [36], containing 182 full-text articles annotated for 21 entities, including elements like treatment dosage and funding institution details. Summerscales et al. [37] similarly annotated a corpus of 263 abstracts, which emphasized treatment groups and outcomes. However, their annotations have a narrower focus, and their corpus remains private.

Wallace et al. [38] offered a solution to the prohibitive costs of constructing large corpora. They used a distant supervision approach to create an extensive full-text article corpus, with 133 articles being manually annotated for evaluation. This method, while cost-effective, raises questions about the quality of the resultant data.

**Figure 5:**
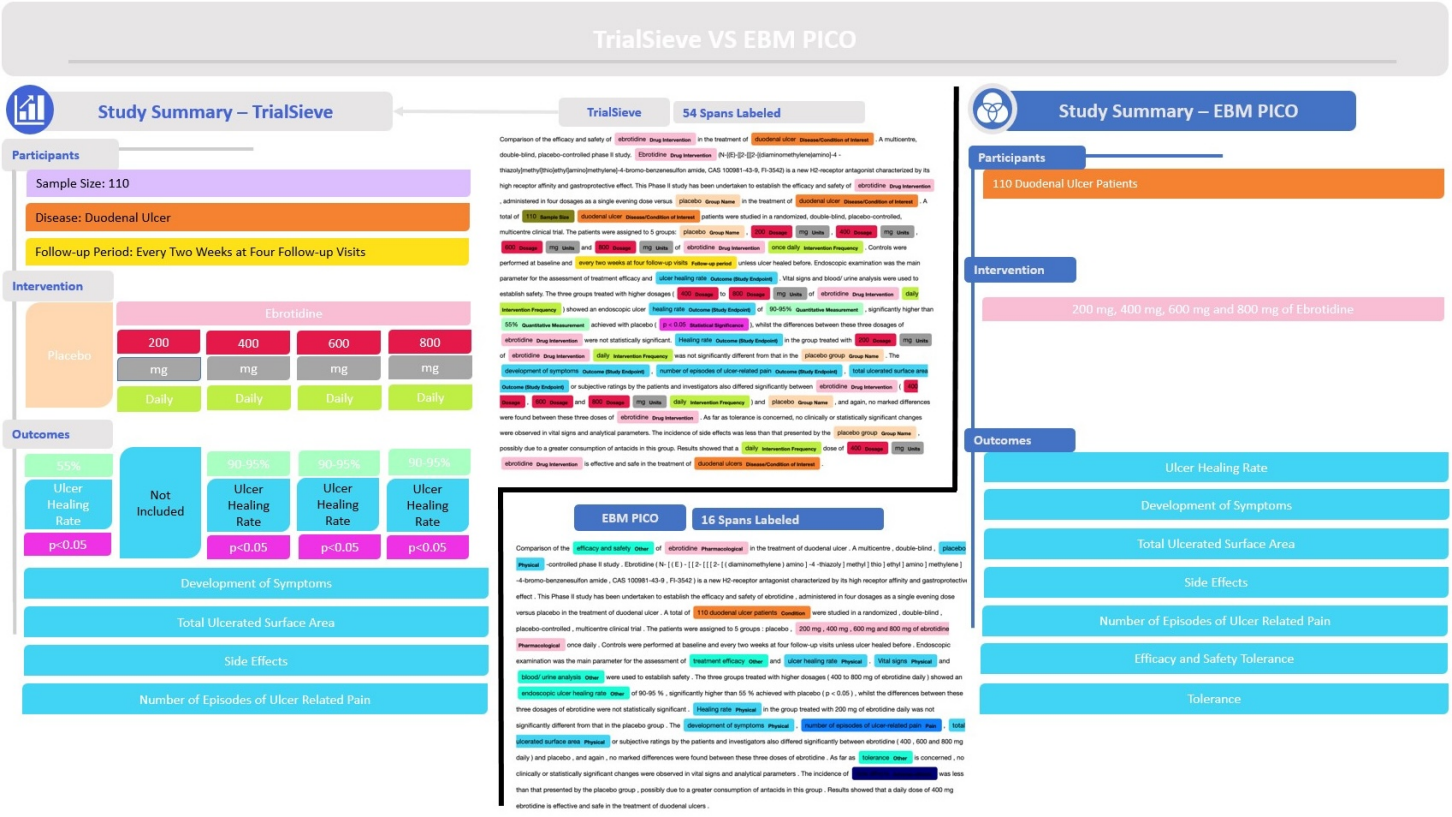
Comparison of annotations in TrialSieve schema versus EBM-PICO [15] on an example biomedical abstract from Gabryelewicz et al. [27]. Previously, patient-intervention-comparator-outcome (PICO) or patient-intervention-outcome (PIO) methods were considered state of the art methods for biomedical information extraction, whether via manual annotation or using automated natural-language processing (NLP) methodologies. The TrialSieve schema results in much more detailed labeling necessary for structured quantitative data information extraction for meta-analysis or other forms of secondary analysis in drug repurposing and/or drug design. In the example abstract, TrialSieve (schema left, example labeled abstract center top) has 54 spans labeled, whereas EBM-PICO (schema right, example labeled abstract center bottom) has only 16 spans labeled.

Most datasets in this field are non-public, with a few notable exceptions like the EBM-NLP corpus by Nye et al. [15], which is one of the largest publicly available corpora. This corpus includes approximately 5000 abstracts from randomized clinical trials (RCTs), primarily for cardiovascular diseases, cancer, and autism. However, it did not include all required fields for meta-analysis, such as numeric texts detailing the number of participants experiencing specific outcomes. The example abstract annotated in Figure 5 shows how the annotations from TrialSieve compare to those of EBM-PICO. In particular, TrialSieve better enables the extraction of data required for quantitative clinical meta-analysis.

In a move towards more comprehensive open-access resources, Mutinda et al. [39] curated a corpus that encapsulates 1011 abstracts from breast cancer RCTs pulled from PubMed. This work primarily focused on the annotation of outcomes. Nonetheless, it is a step forward towards robust evidence-based medicine.

Despite these efforts, there remains a clear need for a comprehensive, publicly accessible biomedical entity dataset with annotations suitable for clinical meta-analysis. TrialSieve aims to address this gap by expanding annotation categories beyond the traditional PICO framework, introducing new annotation aids to enhance human annotation quality and scalability, and providing a comprehensive, open-source annotated dataset. With these advancements, the TrialSieve dataset has the potential to accelerate frontend clinical research and workflows, including quantitative meta-analysis and drug repurposing.

### 4.3. TrialSieve Enables Scalable Annotation by Non-Subject Matter Experts

While the long-term goal is to fully automate biomedical information extraction, some manually annotated datasets are still required for model training and validation. The ability to use non-subject matter experts (e.g., those other than highly specialized clinicians or researchers holding a medical and/or doctoral degree) is one way to increase annotation scalability.

The error analysis (see Table 4) showed that there was a high degree of inter-annotator variability. However, the variance of annotators using the more comprehensive TrialSieve schema was not higher than SMEs [14] using other previous PICO frameworks. The ability to use non-subject matter experts, such as the undergraduate student annotators of the present study, is one way to increase scalability [18].

The annotator survey study found that annotation aids, including the use of motivational aids, relationship trees, and problem-solving sessions, were helpful in increasing non-subject matter expert annotator success. Interestingly, external motivation, such as internal “annotator of the month awards”, were significantly (*p* < 0.05) helpful in increasing morale, productivity, and confidence in accuracy.

### 4.4. Limitations

The TrialSieve dataset annotations were performed by undergraduate students who came from multiple academic disciplines. While all annotators in this study were screened and given thorough training and support to effectively complete the task, there is still disagreement in some annotations that lead to noise in the final dataset.

Additionally, the data consisted of annotated abstracts of scientific articles. Although substantial data are contained in abstracts, some of the elements, particularly quantitative results, are more fully described in the full text of research articles. Future work addressing biomedical information extraction and annotation of full-text articles could provide a more complete picture of the outcomes of each article.

### 4.5. Broader Impacts and Future Directions

While fully automating data extraction for clinical meta-analysis remains a challenge, the TrialSieve framework combined with the state of the NLP or LLM models represents foundational progress. Notably, despite the noisiness of annotated data, BERT-based models trained on such data consistently outperformed most human annotators. The proposed annotation schema can facilitate analyses of various drug-disease, drug-drug, drug-therapy, drug-population, and drug-behavior interactions, positioning TrialSieve as a valuable resource for streamlining frontend clinical research and workflows [40]. Future work should focus on broader testing and implementation, evaluation of relationship extraction, and deploying methods that enable zero-shot annotation by LLMs with accuracies that approach supervised NLP models. Additionally, further evaluation of noise by intentional "poisoning" of annotations to evaluate machine learning defenses will be important for analyzing and improving the robustness and accuracy of automated annotation models despite inevitable human-generated data noise [41].

## 5. Conclusions

TrialSieve introduces a novel biomedical information extraction framework that extends beyond the traditional PICO approach by structuring annotated spans into hierarchical treatment group-based graphs, enabling quantitative outcome comparisons across regimens. TrialSieve was used to annotate 1609 PubMed abstracts with 20 unique entity types relevant to systematic reviews. This publicly available TrialSieve dataset provides an important data resource for biomedical entity recognition projects and related annotation models. The NLP BERT-based models (BioLinkBERT, BioBERT, KRISSBERT, PubMedBERT) and GPT-4o LLM evaluations use TrialSieve as a benchmark to demonstrate that state-of-the-art sequence tagging models can outperform human annotators. Study findings highlight the potential for the TrialSieve framework to improve automated biomedical information extraction that expedites frontend clinical research and streamlines workflows.

## Figures and Tables

**Table 1:** Dataset statistics for dataset annotated using the TrialSieve framework.

Statistic	Value
Num. Abstracts	1609
Num. Words	443,695
Entity Annotations	170,557
Quality Controlled (QC) Annotations	15,854
Distinct Spans	97,004
Relation Annotations	443,695
Distinct Pseudo-Nodes	25,665
Final Spans	52,638

**Table 2:** Aggregated natural-language processing (NLP) model results for automated entity labeling of biomedical abstracts annotated using the TrialSieve schema. Bold font highlights the best performing model for each performance metric.

Model	Accuracy	Precision	Recall	F1-score
BioLinkBERT	**0.875**	0.599	**0.679**	0.637
BioBERT	0.808	0.468	0.574	0.516
KRISSBERT	0.872	0.607	0.658	0.632
PubMedBERT	0.874	**0.614**	0.666	**0.639**
GPT-4o	0.837	0.563	0.459	0.506

**Table 3:** Entity detection results. The performance for each entity type is shown for each model: BioBERT, BioLinkBERT, PubMedBERT, and GPT-4o. Performance metrics included precision (P), recall (R), and F1 score (F1). Bold font highlights the best performing model for each performance metric.

Entity	BioBERT			BioLinkBERT			KRISSBERT			PubMedBERT			GPT-4o		
	P	R	F1	P	R	F1	P	R	F1	P	R	F1	P	R	F1
Disease/Condition of Interest	0.279	0.397	0.328	0.485	0.549	0.515	0.468	0.521	0.494	**0.525**	**0.552**	**0.538**	0.376	0.416	0.395
Dosage	0.502	0.605	0.549	0.754	0.811	0.781	**0.770**	**0.828**	**0.798**	**0.770**	0.795	0.783	0.729	0.729	0.729
Drug Intervention	0.511	**0.653**	0.573	0.578	0.652	**0.613**	0.563	0.603	0.582	0.579	0.609	0.594	**0.603**	0.387	0.471
Follow-up period	0.325	**0.520**	**0.400**	0.312	0.444	0.367	**0.395**	0.333	0.361	0.348	0.356	0.352	0.237	0.517	0.325
Group Characteristic	0.224	0.252	0.238	0.323	**0.392**	0.354	0.301	0.352	0.325	**0.328**	0.390	**0.356**	0.249	0.108	0.151
Group Name	0.293	0.285	0.289	0.393	**0.366**	0.379	0.438	0.344	**0.385**	0.419	0.355	0.384	**0.502**	0.178	0.263
Group Population/Sample Size	0.667	0.801	0.728	**0.828**	**0.849**	**0.838**	0.802	0.835	0.818	0.823	0.836	0.830	0.603	0.387	0.471
Intervention Administration	0.446	0.630	0.523	0.664	**0.652**	0.658	**0.679**	0.643	**0.661**	0.676	0.635	0.655	0.566	0.230	0.327
Intervention Duration	0.535	**0.736**	0.620	0.621	0.685	0.652	0.600	0.685	0.640	**0.636**	0.717	**0.674**	0.551	0.611	0.580
Intervention Frequency	0.614	0.804	0.696	0.679	0.830	0.747	0.689	**0.840**	**0.757**	0.678	**0.840**	0.751	**0.739**	0.364	0.487
Non-Pharmaceutical Intervention	0.059	0.042	0.049	0.143	**0.227**	0.175	0.227	**0.227**	0.227	**0.333**	**0.227**	**0.270**	0.278	0.104	0.151
Non-Study Drug	0.000	0.000	0.000	0.212	0.206	0.209	**0.308**	0.235	**0.267**	0.304	0.206	0.246	0.202	**0.382**	0.264
Outcome (Study Endpoint)	0.279	0.359	0.314	0.423	**0.489**	**0.454**	**0.449**	0.446	0.448	0.418	0.465	0.440	0.193	0.165	0.178
Qualitative Side Effects	0.375	0.316	0.343	**0.462**	0.585	0.516	0.432	0.463	0.447	0.439	0.610	0.510	0.447	**0.732**	**0.555**
Quantitative Measurement	0.611	0.770	0.681	0.686	0.819	0.747	0.687	0.824	0.749	**0.699**	**0.828**	**0.758**	0.606	0.719	0.657
Statistical Significance	0.746	0.825	0.784	0.882	**0.956**	**0.918**	0.828	0.953	0.886	0.890	0.947	**0.918**	**0.930**	0.852	0.889
Study Duration	0.462	0.500	0.480	0.444	**0.667**	0.533	0.412	0.583	0.483	**0.500**	**0.667**	**0.571**	0.289	0.243	0.264
Study Years	0.280	0.333	0.304	0.500	**0.821**	0.622	0.525	0.750	0.618	**0.538**	0.750	**0.627**	0.500	0.485	0.492
Type of Quant. Measure	0.277	0.468	0.348	0.479	0.716	0.574	0.502	**0.720**	0.591	**0.507**	**0.720**	**0.595**	0.434	0.219	0.291
Units	0.499	0.641	0.561	0.715	**0.787**	0.749	0.730	0.754	0.741	**0.750**	0.758	**0.754**	0.681	0.341	0.454

**Table 4:** Cleaned Error Analysis.

Annotation Source	Total Mentions Annotated	Correct Annotations	Errors
BioLinkBERT	3103	2218 (71.48%)	885 (28.52%)
Human	2463	1240 (50.35%)	1223 (49.65%)

**Table 5:** Type of errors.

Annotation Source	Wrong Label	Wrong Overlap	Multiple Entities	Invalid Tag
BioLinkBERT	302 (34.12%)	382 (43.16%)	41 (4.63%)	160 (18.08%)
Human	481 (39.33%)	509 (41.62%)	79 (6.46%)	154 (12.59%)

**Table 6:** TrialSieve significant findings from the annotator user study. An anonymous survey was deployed to examine annotator opinions on motivational aids, the use of relationship tress, and team annotation sessions. A total of 38 of the 72 annotators in the study completed the voluntary survey. A standard 1 to 5 Likert scale was utilized where one was “strongly agree” and 5 was “strongly disagree”. Mean, standard deviation (SD), and *p*-value is shown. Significance/insignificance was based on a standard alpha of 0.05. Survey questions with insignificant *p*-values are shown in Table A1.

Aid	Survey Question	Mean	SD	*p*-Value
**Motivational Aids**	The potential to be awarded the “Annotator of the Month” award made me strive to be more accurate with my annotations	2.324	1.065	<0.01
	I was motivated to annotate as accurately as possible for the purpose of attaining the grade I desire and course credit for research	2.152	1.228	<0.01
	Having the chance to be promoted to the leadership team motivated me to complete my tasks more accurately/efficiently	2.063	1.190	<0.01
	Technical issues with the curation software made me less motivated to try and complete my annotations accurately	2.571	0.815	<0.01
**Relationship Trees**	Relationship trees have overall been a useful tool for me to label text	1.824	0.999	<0.01
	I found that using relationship trees made it easier for me to label text accurately	2.057	1.027	<0.01
	I believe I may have made LESS errors when I used relationship trees when annotating	1.971	0.870	<0.01
	Using relationship trees improves my ability to annotate accurately	1.912	0.933	<0.01
	I would prefer to label using relationship trees	2.457	1.172	<0.05
**Team Sessions**	Collaborating with teammates to label abstracts during in-person and virtual sessions made me understand how to label abstracts more accurately	2.394	1.029	<0.01

## Data Availability

The annotated TrialSieve dataset is publicly available at https://github.com/pathology-dynamics/trialsieve_final [accessed 26 April 2025]. The code for the open-source NLP annotation software, LightTag, can be found at https://github.com/LightTag [accessed 26 April 2025].

## Abbreviations

The following abbreviations are used in this manuscript:

PICO	Patient Intervention Comparator Outcome
NLP	Natural-Language Processing
BioNER	Biomedical Named Entity Recognition
BERT	Bidirectional Encoder Representations from Transformers
DPI	Multidisciplinary Digital Publishing Institute
DOAJ	Directory of open-access journals

## Appendix A

**Table A1:** Insignificant findings from the annotator user study. An anonymous survey was deployed to examine annotator opinions on motivational aids, the use of relationship tress, and team annotation sessions. A total of 38 of the 72 annotators in the study completed the voluntary survey. A standard 1 to 5 Likert scale was utilized where one was “strongly agree” and 5 was “strongly disagree”. Mean, standard deviation (SD), and *p*-value is shown. Significance/insignificance was based on a standard alpha of 0.05. Remaining survey questions (with significant *p*-value) are shown in Table 6.

Aid	Survey Question	Mean	SD	α
Motivational Aids	The “Curator of the Month” award was not something I considered when I made annotations	3.059	1.301	0.602
	My grade for this course was not something I thought about when I made annotations	2.882	1.320	0.303
	I did not think about potential team promotions when I made my annotations	2.806	1.238	0.176
	Technical issues with the curation software were not something that affected my accuracy	3.250	1.180	0.893
Relationship Trees	I found that using relationship trees made it more difficult for me to label text	3.750	1.016	0.999
	Relationship trees have added stress for me when labeling texts	3.114	1.132	0.723
Team Sessions	I felt that the written dictionary/annotation guides were just as effective as the in-person/virtual sessions	3.000	1.163	0.500
	I believe the in-person/virtual sessions helped me make less errors	3.235	1.161	0.872

## References

[1] Hobbs J.R. (2002). Information extraction from biomedical text. J. Biomed. Inform..

[2] Wang Y., Wang L., Rastegar-Mojarad M., Moon S., Shen F., Afzal N., Liu S., Zeng Y., Mehrabi S., Sohn S. (2018). Clinical information extraction applications: A literature review. J. Biomed. Inform..

[3] Prasad V., Mailankody S. (2017). Research and development spending to bring a single cancer drug to market and revenues after approval. JAMA Intern. Med..

[4] Wouters O.J., McKee M., Luyten J. (2020). Estimated research and development investment needed to bring a new medicine to market, 2009–2018. JAMA.

[5] Chong C.R., Sullivan D.J. (2007). New uses for old drugs. Nature.

[6] Bakowski M.A., Beutler N., Wolff K.C., Kirkpatrick M.G., Chen E., Nguyen T.T.H., Riva L., Shaabani N., Parren M., Ricketts J. (2021). Drug repurposing screens identify chemical entities for the development of COVID-19 interventions. Nat. Commun..

[7] Masoudi-Sobhanzadeh Y., Omidi Y., Amanlou M., Masoudi-Nejad A. (2020). Drug databases and their contributions to drug repurposing. Genomics.

[8] Ashburn T.T., Thor K.B. (2004). Drug repositioning: Identifying and developing new uses for existing drugs. Nat. Rev. Drug Discov..

[9] Kim H., Kang J. (2022). How do your biomedical named entity recognition models generalize to novel entities?. IEEE Access.

[10] Schiavenato M., Chu F. (2021). PICO: What it is and what it is not. Nurse Educ. Pract..

[11] Muka T., Glisic M., Milic J., Verhoog S., Bohlius J., Bramer W., Chowdhury R., Franco O.H. (2020). A 24-step guide on how to design, conduct, and successfully publish a systematic review and meta-analysis in medical research. Eur. J. Epidemiol..

[12] Borah R., Brown A.W., Capers P.L., Kaiser K.A. (2017). Analysis of the time and workers needed to conduct systematic reviews of medical interventions using data from the PROSPERO registry. BMJ Open.

[13] Al-Hussaini I., Nakajima An D., Lee A.J., Bi S., Mitchell C.S. CCS Explorer: Relevance Prediction, Extractive Summarization, and Named Entity Recognition from Clinical Cohort Studies. Proceedings of the 2022 IEEE International Conference on Big Data (Big Data).

[14] Kartchner D., Ramalingam S., Al-Hussaini I., Kronick O., Mitchell C. Zero-Shot Information Extraction for Clinical Meta-Analysis using Large Language Models. Proceedings of the the 22nd Workshop on Biomedical Natural Language Processing and BioNLP Shared Tasks.

[15] Nye B., Li J.J., Patel R., Yang Y., Marshall I., Nenkova A., Wallace B. A Corpus with Multi-Level Annotations of Patients, Interventions and Outcomes to Support Language Processing for Medical Literature. Proceedings of the 56th Annual Meeting of the Association for Computational Linguistics (Volume 1: Long Papers).

[16] Lee J., Yoon W., Kim S., Kim D., Kim S., So C.H., Kang J. (2020). BioBERT: A pre-trained biomedical language representation model for biomedical text mining. Bioinformatics.

[17] Gu Y., Tinn R., Cheng H., Lucas M., Usuyama N., Liu X., Naumann T., Gao J., Poon H. (2020). Domain-Specific Language Model Pretraining for Biomedical Natural Language Processing. arXiv.

[18] Mitchell C.S., Cates A., Kim R.B., Hollinger S.K. (2015). Undergraduate biocuration: Developing tomorrow’s researchers while mining today’s data. J. Undergrad. Neurosci. Educ..

[19] Artstein R. (2017). Inter-annotator Agreement. Handbook of Linguistic Annotation.

[20] Newman-Griffis D., Lehman J.F., Rosé C., Hochheiser H. Translational NLP: A New Paradigm and General Principles for Natural Language Processing Research. Proceedings of the 2021 Conference of the North American Chapter of the Association for Computational Linguistics: Human Language Technologies.

[21] Sanchez-Graillet O., Witte C., Grimm F., Cimiano P. (2022). An annotated corpus of clinical trial publications supporting schema-based relational information extraction. J. Biomed. Semant..

[22] Kartchner D., Al-Hussaini I., Haydn T., Deng J., Lohiya S., Bathala P., Mitchell C. BioSift: A Dataset for Filtering Biomedical Abstracts for Drug Repurposing and Clinical Meta-Analysis. Proceedings of the 46th International ACM SIGIR Conference on Research and Development in Information Retrieval (SIGIR).

[23] Perry T. LightTag: Text Annotation Platform. Proceedings of the 2021 Conference on Empirical Methods in Natural Language Processing: System Demonstrations.

[24] Collins L.M. (2007). Research Design and Methods. Encyclopedia of Gerontology.

[25] Lee G.E., Sun A. A Study on Agreement in PICO Span Annotations. Proceedings of the 42nd International ACM SIGIR Conference on Research and Development in Information Retrieval.

[26] Loshchilov I., Hutter F. (2017). Decoupled weight decay regularization. arXiv.

[27] Gabryelewicz A., Czajkowski A., Skrodzka D., Marlicz K., Luca de Tena F., Aldeguer M., Chantar C., Márquez M., Torres J., Ortiz J. (1997). Comparison of the efficacy and safety of ebrotidine in the treatment of duodenal ulcer. A multicentre, double-blind, placebo-controlled phase II study. Arzneimittel-Forschung.

[28] Langer H., Lungen H., Bayerl P.S. Text Type Structure and Logical Document Structure. Proceedings of the Workshop on Discourse Annotation.

[29] Garcia-Retamero R., Hoffrage U. (2013). Visual representation of statistical information improves diagnostic inferences in doctors and their patients. Soc. Sci. Med..

[30] Hu Y., Keloth V.K., Raja K., Chen Y., Xu H. (2023). Towards precise PICO extraction from abstracts of randomized controlled trials using a section-specific learning approach. Bioinformatics.

[31] Ge T., Luo X., Wang Y., Sedlmair M., Cheng Z., Zhao Y., Liu X., Deussen O., Chen B. (2024). Optimally ordered orthogonal neighbor joining trees for hierarchical cluster analysis. IEEE Trans. Vis. Comput. Graph..

[32] Woloshin S., Yang Y., Fischhoff B. (2023). Communicating health information with visual displays. Nat. Med..

[33] Finson K., Pederson J. (2011). What are Visual Data and What Utility do they have in Science Education?. J. Vis. Lit..

[34] Arain S.A., Afsar N.A., Rohra D.K., Zafar M. (2019). Learning clinical skills through audiovisual aids embedded in electronic-PBL sessions in undergraduate medical curriculum: Perception and performance. Adv. Physiol. Educ..

[35] Naqvi S.H., Mobasher F., Afzal M.A.R., Umair M., Kohli A.N., Bukhari M.H. (2013). Effectiveness of teaching methods in a medical institute: Perceptions of medical students to teaching aids. J. Pak. Med. Assoc..

[36] Kiritchenko S., De Bruijn B., Carini S., Martin J., Sim I. (2010). ExaCT: Automatic extraction of clinical trial characteristics from journal publications. BMC Med. Inform. Decis. Mak..

[37] Summerscales R.L., Argamon S., Bai S., Hupert J., Schwartz A. Automatic summarization of results from clinical trials. Proceedings of the 2011 IEEE International Conference on Bioinformatics and Biomedicine.

[38] Wallace B.C., Kuiper J., Sharma A., Zhu M., Marshall I.J. (2016). Extracting PICO sentences from clinical trial reports using supervised distant supervision. J. Mach. Learn. Res..

[39] Mutinda F., Liew K., Yada S., Wakamiya S., Aramaki E. PICO Corpus: A Publicly Available Corpus to Support Automatic Data Extraction from Biomedical Literature. Proceedings of the First Workshop on Information Extraction from Scientific Publications.

[40] Nia A.M., Mozaffari-Kermani M., Sur-Kolay S., Raghunathan A., Jha N.K. (2015). Energy-Efficient Long-term Continuous Personal Health Monitoring. IEEE Trans.-Multi-Scale Comput. Syst..

[41] Mozaffari-Kermani M., Sur-Kolay S., Raghunathan A., Jha N.K. (2015). Systematic Poisoning Attacks on and Defenses for Machine Learning in Healthcare. IEEE J. Biomed. Health Inform..

